# An *Arabidopsis* Mutant Over-Expressing Subtilase SBT4.13 Uncovers the Role of Oxidative Stress in the Inhibition of Growth by Intracellular Acidification

**DOI:** 10.3390/ijms21031173

**Published:** 2020-02-10

**Authors:** Gaetano Bissoli, Jesús Muñoz-Bertomeu, Eduardo Bueso, Enric Sayas, Edgardo A. Vilcara, Amelia Felipo, Regina Niñoles, Lourdes Rubio, José A. Fernández, Ramón Serrano

**Affiliations:** 1 Instituto de Biología Molecular y Celular de Plantas, Universidad Politécnica de Valencia-Consejo Superior de Investigaciones Científicas, Camino de Vera, 46022 Valencia, Spain; gaetano.bissoli@mail.com (G.B.); edbuero@ibmcp.upv.es (E.B.); ensamon@upvnet.upv.es (E.S.); edavilcara@gmail.com (E.A.V.); afelipo@gmail.com (A.F.); renioro@upvnet.upv.es (R.N.); 2 Departament de Biologia Vegetal, Facultat de Farmàcia, Universitat de València, 46100 València, Spain; jesus.munoz-bertomeu@uv.es; 3 Departamento de Botánica y Fisiología Vegetal, Universidad de Málaga, 29071 Málaga, Spain; lrubio@uma.es (L.R.); ja_fernandez@uma.es (J.A.F.)

**Keywords:** activation-tagging, organic acids, H^+^-ATPase, NADPH oxidase, ROS

## Abstract

Intracellular acid stress inhibits plant growth by unknown mechanisms and it occurs in acidic soils and as consequence of other stresses. In order to identify mechanisms of acid toxicity, we screened activation-tagging lines of *Arabidopsis thaliana* for tolerance to intracellular acidification induced by organic acids. A dominant mutant, *sbt4.13-1D*, was isolated twice and shown to over-express subtilase SBT4.13, a protease secreted into endoplasmic reticulum. Activity measurements and immuno-detection indicate that the mutant contains less plasma membrane H^+^-ATPase (PMA) than wild type, explaining the small size, electrical depolarization and decreased cytosolic pH of the mutant but not organic acid tolerance. Addition of acetic acid to wild-type plantlets induces production of ROS (Reactive Oxygen Species) measured by dichlorodihydrofluorescein diacetate. Acid-induced ROS production is greatly decreased in *sbt4.13-1D* and *atrboh-D,F* mutants. The latter is deficient in two major NADPH oxidases (NOXs) and is tolerant to organic acids. These results suggest that intracellular acidification activates NOXs and the resulting oxidative stress is important for inhibition of growth. The inhibition of acid-activated NOXs in the *sbt4.13-1D* mutant compensates inhibition of PMA to increase acid tolerance.

## 1. Introduction

Slightly alkaline cytosolic pH (pHc) is a universal feature of actively growing eukaryotic cells [1]. On the other hand, intracellular acidification inhibits cell growth [1] and, if intense enough, triggers cell death [2,3,4]. The molecular mechanisms of regulation of cell growth and death by pHc are mostly unknown and constitute a fundamental problem in biology.

Intracellular acidification is especially relevant for plant physiology because it is the consequence of many abiotic stresses, such as acidic media [5], heat shock [6,7] and K^+^ starvation [8]. Also, fermentations under hypoxic conditions produce protons in the oxidation of sugars to organic acids [9,10]. In this respect, carboxylation/decarboxylation reactions have been proposed to constitute a biochemical pH-stat stabilizing pHc [11]. The plasma membrane H^+^-ATPase (PMA) and the vacuolar H^+^-ATPase and H^+^-PPase are responsible for the extrusion of protons from the cytosol and constitute a biophysical pH-stat. The activities of these proton pumps are inhibited under energy deprivation, a condition prevailing during many stresses, and then pHc decreases [12]. In addition, during water stress abscisic acid increases and inhibits PMA to produce intracellular acidification and growth inhibition [13].

In plants most attention has been paid to extracellular pH (pHe) because of the toxicity of acidic soils. In these conditions aluminum toxicity comes into operation because of solubilization of this toxic cation at low pH. A zinc finger transcription factor known as STOP1 regulates multiple genes that protect growth of *Arabidopsis thaliana* roots from low pHe and aluminum [5,14]. STOP1 is conserved in other plants and it activates genes concerned with K^+^ uptake and with synthesis and efflux of malate and citrate to sequester aluminum [14,15].

Although acid pHe lowers pHc, changing pHe is not the best way to alter pHc because other cellular parameters are affected by acidic pHe. We have developed a different experimental approach based on the utilization of weak organic acids (acetic, propionic, sorbic) to induce intracellular acidification without altering pHe. When buffered pHe is close to the pKa of the acid, the protonated form diffuses into cells and acidifies the cytosol. This results in inhibition of germination and seedling establishment (expanded green cotyledons). At the low acid concentrations used in our *Arabidopsis* experiments this inhibition of growth is not accompanied by death of germinating seeds and it just delays seedling establishment by a few days [16]. To further validate our intracellular acidification assay, we have demonstrated sensitivity to organic acids of mutants with loss of function of major root K^+^ channel (AKT1), tonoplast dicarboxylate (malate) transporter (TDT), vacuolar H^+^-ATPase and vacuolar H^+^-Na^+^/K^+^ antiporter NHX2 [16].

We have previously described two *Arabidopsis* mutants more tolerant to organic acids: *rof2-1D*, over-expressing ROF2 prolyl isomerase [17] and *wat1-1D*, a dominant-negative form of AP-3 adaptin [16]. In both mutants K^+^ uptake is increased, resulting in plasma membrane depolarization and consequent activation of the electrogenic plasma membrane H^+^-ATPase to reduce intracellular acidification. Interestingly, the transcription factor STOP1 described above induces genes related to K^+^ uptake, such as *CIPK23*, encoding a protein kinase that activates the root K^+^ channel AKT1, and *HAK5*, encoding a high affinity K^+^ transporter [14,15]. However, transcriptomic studies indicate that genes regulated by organic acids at normal pHe [17] are different from those regulated by acid pHe [14,15].

In the present work we describe a novel mutant tolerant to organic acids that, surprisingly, does not reduce intracellular acidification induced by these acids and under normal conditions has a lower pHc than control. The mutation is caused by over-expression of the endoplasmic reticulum protease SBT4.13. This results in decreased activity of PMA because of partial degradation of the enzyme, a change that cannot explain acid tolerance but explains other phenotypic features of the mutant. We have observed that a decrease of pHc by organic acids induces production of ROS (Reactive Oxygen Species) dependent on two major NADPH oxidases (NOXs and RBOHs, or Respiratory Burst Oxidase Homologues) of *Arabidopsis* (AtRBOH-D and AtRBOH-F). This activation is greatly reduced in the mutant over-expressing the protease. These results suggest that one mediator of growth inhibition by intracellular acidification is the production of ROS by activation of NOXs and that over-expression of protease SBT4.13 inhibits PMA and acid activation of NOX.

## 2. Results

### 2.1. A Novel Mutant Tolerant to Weak Organic Acids Over-Expresses Subtilase SBT4.13

**Figure 1:**
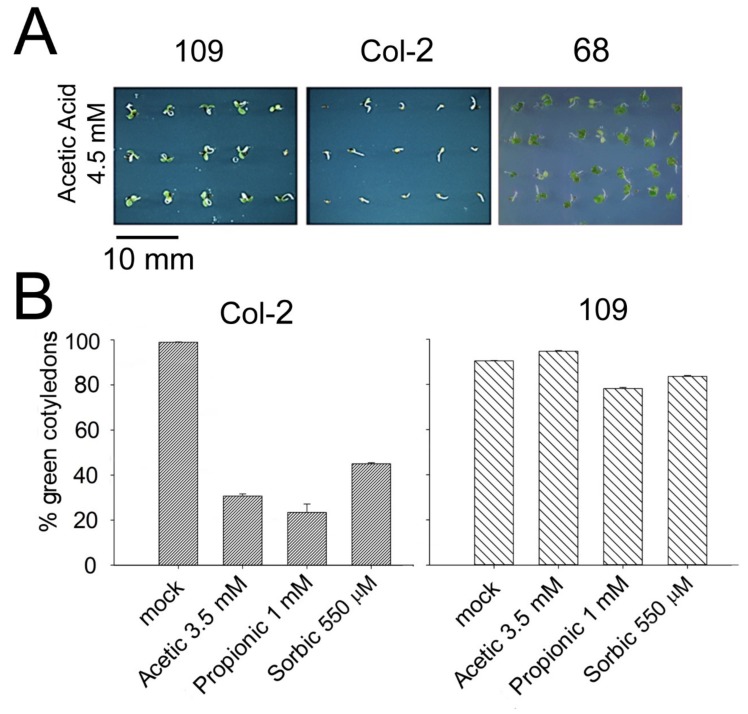
Acid tolerance of two mutants isolated from the screening of the W. Sheible and C. Sommerville activation-tagging collection and having a single T-DNA insertion by Southern analysis (see Figure S1). (**A**) Germination and seedling establishment (seedlings with green and expanded cotyledons) of wild type (Col2 ecotype of *Arabidopsis thaliana*) and two derived mutants (coded 68 and 109). Picture was taken six days after sowing in MS medium buffered at pH 5.5 and supplemented with 4.5 mM acetic acid. Seedling establishment was more than 95% in the absence of acids (see mock in panel B). (**B**) Statistical data from three independent experiments like the one in **A** (20–30 seedlings from every line in each experiment) with wild type and mutant 109 in media without acids (mock) and with three different organic acids at the indicated concentrations. Error bars correspond to standard error. All differences with wild type are statistically significant with *p* < 0.01 by Student’s t-test.

We have screened 63,000 lines of an “activation tagging” mutant seed collection (about 600,000 seeds) looking for improved tolerance to organic acids during germination and seedling establishment (appearance of green cotyledons). From 21 confirmed mutants we identified two (coded 68 and 109) with apparently the same single T-DNA insertion as determined by Southern analysis (Figure S1). The progeny of these lines were 100% resistant to BASTA and therefore they were homozygous for T-DNA. The acid phenotype of these mutants is shown in Figure 1A. These mutants are more tolerant than wild type to three weak organic acids of similar pKa (4.75–4.9): acetic, propionic and sorbic (Figure 1B). Therefore, their phenotype is probably related to intracellular acidification produced by the acids and not to other features of the molecules.

**Figure 2:**
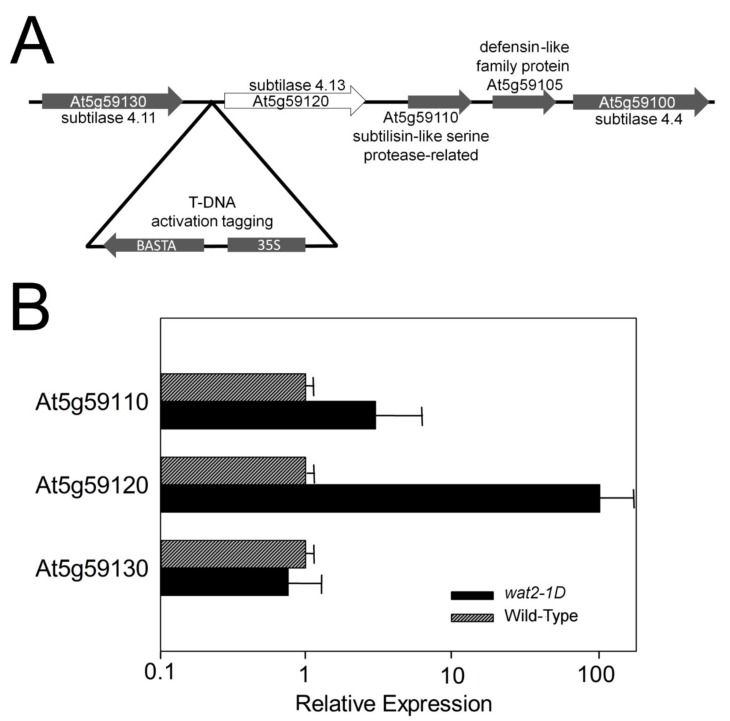
Molecular characterization of *wat2-1D*, a novel mutant tolerant to weak organic acids. (**A**) Position of the T-DNA in chromosome V of the genome of the mutant as determined by plasmid rescue and sequencing. The minus strand of a genomic region within coordinates 23873849 to 23858873 (about 15 kb) is shown. Introns are not detailed. T-DNA is not to scale because it would be too small. (**B**) Expression analysis by qRT-PCR of genes close to the inserted 35S enhancer (labeled as 35S in part **A**). Values are in logarithmic scale and relative to expression of wild type taken as 1. Error bars correspond to standard error. Only in the case of At5g59120 difference with wild type is statistically significant with *p* < 0.01 by Student’s t-test.

Plasmid rescue of the genomic region containing the T-DNA insertion and sequencing indicates that these two mutants contained the same insertion at chromosome V (Figure 2A). Crossing with wild type and analysis of acetic acid tolerance in F2 demonstrated that the mutation was monogenic and dominant (segregates 3:1). From 357 F2 seeds sowed in media with 5.5 mM acetic acid, 267 were tolerant and 89 sensitive (the chi-square value was 0.79, much lower than the 3.84 value for *p* = 0.05). Analysis by PCR of 32 acid-tolerant seedlings indicates that all contained T-DNA (Figure S2). We named this novel mutant *wat2-1D* (from weak acid tolerant 2, the *wat1-1D* mutant is the one of reference [16]). *1D* means “allele 1, Dominant.”

The genomic region of the T-DNA insertion contains three genes with introns encoding proteases of the subtilase family (subtilisin-like or SBTs): At5g59130 (*SBT4.11*), At5g59120 (*SBT4.13*) and At5g59110 (subtilisin-like protein). The 35S enhancer of T-DNA is close (0.58 kb) to the beginning of the *SBT4.13* gene and analysis of gene expression in the mutant as compared to wild type indicates that this gene is the only one greatly over-expressed in the mutant (about 100-fold; Figure 2B). The At5g59110 gene is slightly more expressed in the mutant (3–6 fold) but the difference with wild type is not statistically significant. Therefore, our hypothesis at this stage was that the acetic acid-tolerance mutation obtained by activation tagging is caused by over-expression of the *SBT4.13* gene.

### 2.2. Recapitulation of Phenotypic Features of the Wat2-1D Mutant by Transgenic Plants Over-Expressing the SBT4.13 Gene

**Figure 3:**
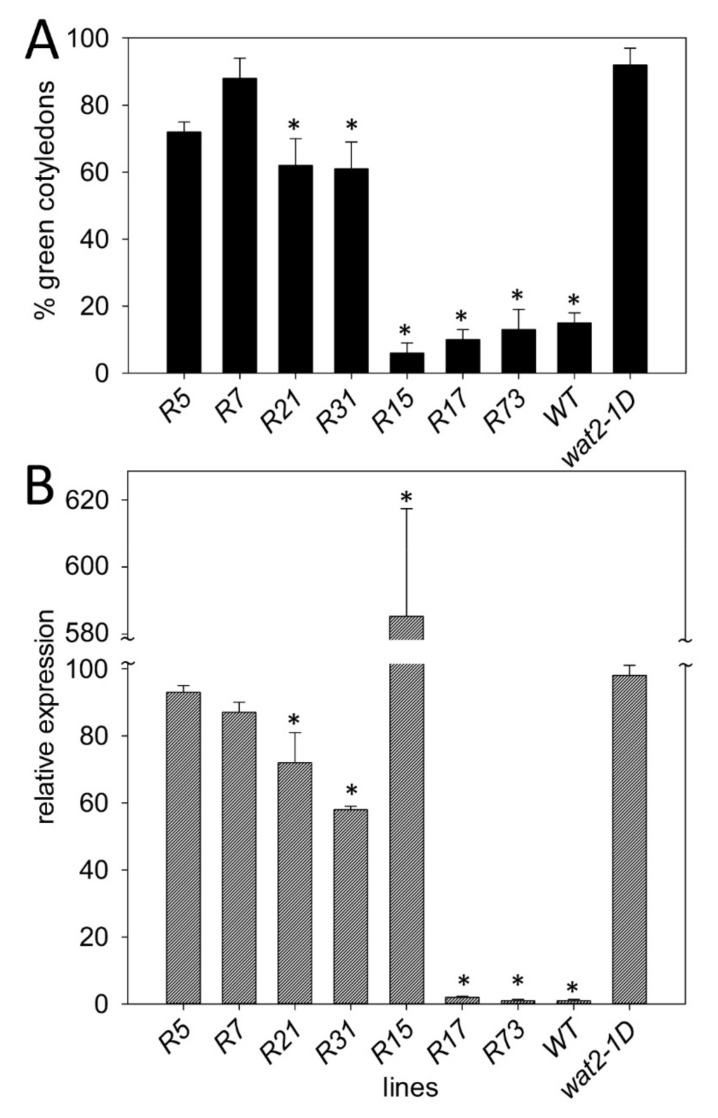
Recapitulation of tolerance to acetic acid in transgenic lines over-expressing *SBT4.13*. (**A**) Percentage of seedlings with green and expanded cotyledons in wild type (Col2), *wat2-1D* mutant and seven transgenic lines (coded R5, R7, R21, R31, R15, R17 and R73), six days after sowing in MS medium buffered at pH 5.5 and supplemented with 3.5 mM acetic acid. (**B**) Relative expression of *SBT4.13* in wild type, *wat2-1D* mutant and seven transgenic lines as in A. Expression in wild type is taken as 1. Values are the average of three independent experiments and error bars correspond to standard error. Asterisks indicate that differences with *wat2-1D* are statistically significant with *p* < 0.01 by Student’s t-test.

To confirm the relationship between over-expression of *SBT4.13* and tolerance to weak organic acids we recapitulated this phenotypic feature in transgenic plants transformed with a T-DNA containing the genomic coding region of this gene cloned under control of the 35S promoter. Seven homozygous transgenic lines were isolated and analyzed for acetic acid tolerance and expression of *SBT4.13*. Four of them recapitulated the acetic acid tolerance of *wat2-1D* and the other three lines showed no acid tolerance (Figure 3A). As indicated in Figure 3B, acid tolerance seems to require over-expression of *SBT4.13* (60 to 100-fold) in the range of the *wat2-1D* mutant. Of three negative lines, one over-expresses too much (more than 500-fold) and two over-express too little (less than-2 fold). The best two lines (coded R5 and R7, R for Recapitulation line) were selected for further studies. In normal medium without acid all lines exhibited more than 95% plantlets with green cotyledons (see Figure S3 for wild type, *wat2-1D* and R5 and R7 lines).

**Figure 4:**
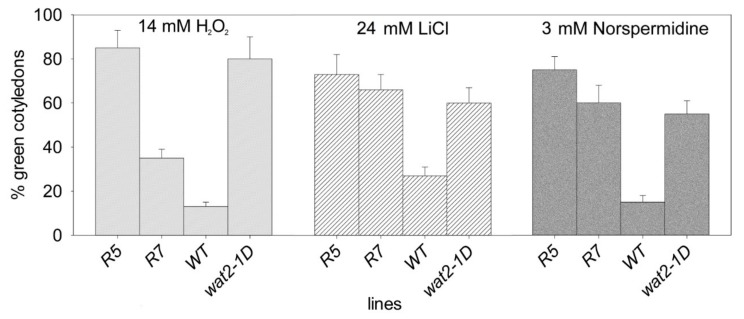
Recapitulation of tolerance to H_2_O_2_ and toxic cations (lithium and norspermidine) in transgenic lines over-expressing *SBT4.13*. Percentage of seedlings with green and expanded cotyledons in wild type (Col2), *wat2-1D* mutant and transgenic lines over-expressing *SBT4.13* (R5 and R7), six days after sowing in MS medium supplemented with the indicated concentrations of toxicants. Statistical data from three independent experiments with 25–35 seedlings from every line are shown. Error bars correspond to standard error and all differences with wild type are statistically significant with *p* < 0.01 by Student’s t-test. In normal medium without toxicants all lines exhibited more than 95% plantlets with green cotyledons (see Figure S3).

**Figure 5:**
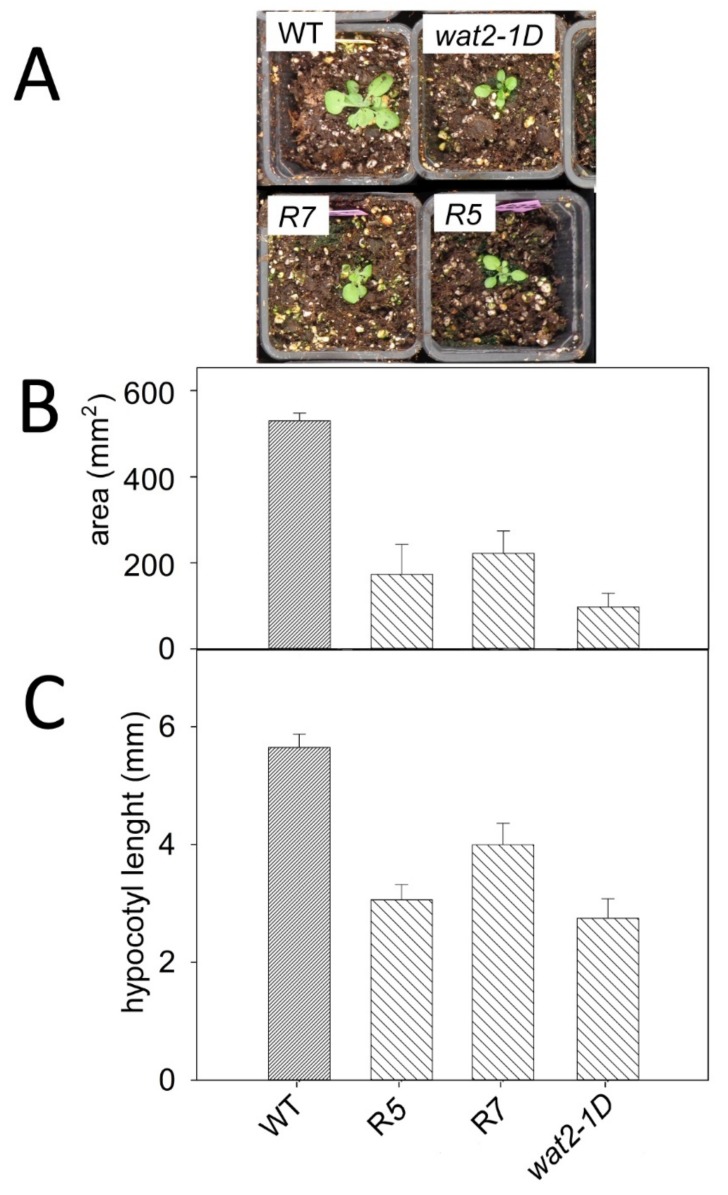
Recapitulation of the small size feature in transgenic lines over-expressing *SBT4.13*. (**A**) Aspect of representative plantlets two weeks after sowing in soil. (**B**,**C**) Statistical data from three independent experiments with 25–35 seedlings are shown. Error bars correspond to standard error and all differences with wild type are statistically significant with *p* < 0.01 by Student’s t-test.

The transgenic plants (R5 and R7) also recapitulate additional phenotypic features of *wat2-1D* mutant, such as tolerance to hydrogen peroxide, lithium and norspermidine (Figure 4) and smaller plant size (Figure 5). Therefore, the *wat2-1D* mutant was renamed as *sbt4.13-1D* and the R5 line was selected as representative for further experiments.

### 2.3. Electrophysiological Characterization of the sbt4.13-1D Mutant

**Figure 6:**
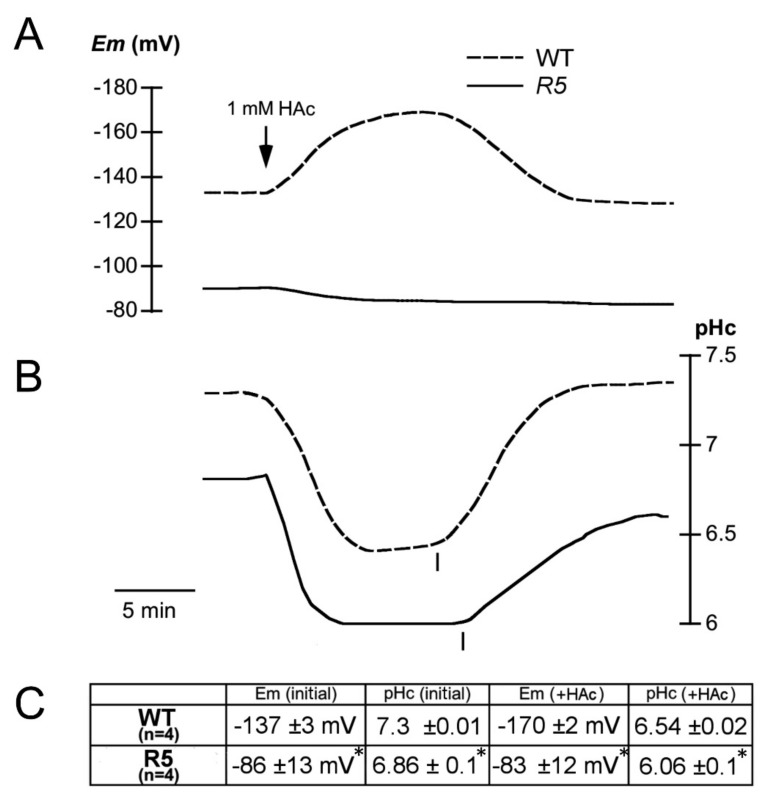
Electrophysiological analysis of root epidermal cells in wild type (WT) and the *sbt4.13-1D* over-expression mutant R5. (**A**) Plasma membrane electrical potential (Em, in millivolts) of wild type (Col2 ecotype of *Arabidopsis thaliana*; discontinuous line) and R5 line recapitulating the *sbt4.13-1D* mutant (continuous line). (**B**) Simultaneous cytosolic pH (pHc) measurements in the same cells with treatments as in A. Acetic acid (1 mM final) addition is indicated by the arrow and the removal by a vertical line in both A and B. (**C**) statistical data from four determinations of Em and pHc (average ± standard error). For each variable asterisks indicate that differences with wild type are statistically significant with *p* < 0.01 by Student’s t-test.

In order to determine if acid tolerance is caused by improved pHc homeostasis, electrophysiological studies were performed in root epidermal cells of seedlings two weeks old [13,16,17]. As indicated in Figure 6A,C, plasma membranes of mutant root cells were depolarized as compared to wild type (−86 versus −137 mV). This depolarization would explain tolerance to toxic cations such as lithium and norspermidine because the electrical potential contributes to the driving force for cation uptake [17,18]. Addition of acetic acid induces a reversible hyperpolarization in wild type (Figure 6A,C), explained by activation of PMA by intracellular acidification [16,19]. Of note, this hyperpolarization does not occur in the mutant. Also, to our surprise, pHc was more acidic in the mutant, both under normal conditions (6.86 versus 7.30) and in the presence of acetic acid (6.06 versus 6.54) and recovery from a pulse of acetic acid was slower than in wild type (Figure 6B,C).

These results suggest a lower activity of PMA in the mutant, something unexpected in plants tolerant to intracellular acidification and at variance with previous acid-tolerant mutants. In the *rof2-1D* [17] and *wat1-1D* [16] mutants, activation of K^+^ uptake causes a depolarization that increases H^+^ efflux mediated by the electrogenic PMA. Accordingly, these mutants exhibit pHc in the presence of acetic acid higher than wild type and recovery of pHc after a pulse of acetic acid was faster than in wild type. Also, intracellular K^+^ during normal growth was similar to wild type in previous mutants while the *sbt4.13-1D* mutant has a lower K^+^ content than wild type (0.76 ± 0.05 versus 1.10 ± 0.04 µmoles/mg dry weight; *n* = 3; difference is significant with *p* < 0.01 by Student’s t-test).

Clearly the *sbt4.13-1D* mutant has K^+^ and pHc homeostasis less robust than wild type and therefore it is difficult to explain its tolerance to weak organic acids by the same mechanism operating in previous acid tolerant mutants.

### 2.4. The sbt4.13-1D Mutant is Defective in Plasma Membrane H^+^-ATPase

**Figure 7:**
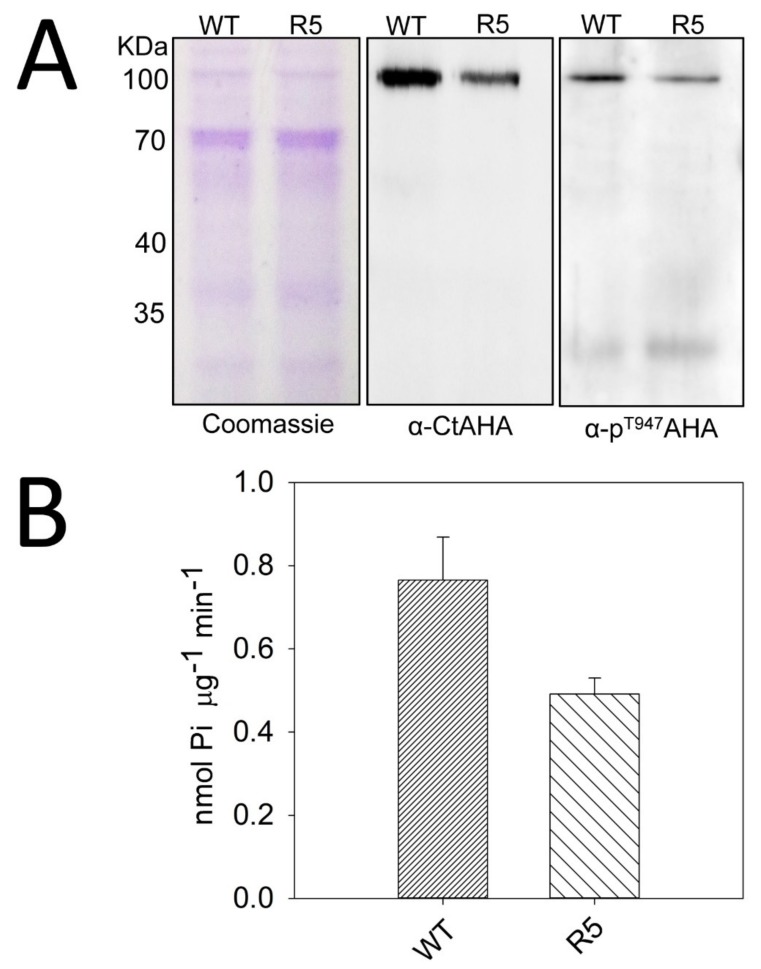
The *sbt4.13-1D* mutant contains less PMA. (**A**) Western immuno-blot analysis of partially purified plasma membrane vesicles shows less PMA protein (α-CtAHA, antibody against carboxy-terminal domain) and less active PMA (phosphorylated in penultimate threonin, antibody α-p^T947^) in *sbt4.13-1D* mutant (line R5) than in wild type. The left panel shows a gel stained for total proteins with Coomassie R-250. (**B**) Measurement of PMA activity in partially purified plasma membrane vesicles shows less activity in *sbt4.13-1D* mutant (line R5) than in wild type. The experiment has been repeated three times with similar results and error bars in B correspond to standard error. Differences with wild type are statistically significant with *p* < 0.01 by Student’s t-test.

We investigated the probable lower activity of PMA in the *sbt4.13-1D* mutant by partial purification of plasma membrane vesicles and determination of PMA amount by Western blot analysis with two antibodies that recognize the three major isoforms (AHA1, AHA2 and AHA3) [13]. One recognizes the whole C-terminal domain (98 amino acids, α-CtAHA) and another the phosphorylated peptide at the end of C-terminus (last nine amino acids, penultimate threonine phosphorylated, α-p^T947^). The latter phosphorylation correlates with an activated state of PMA. As indicated in Figure 7A, with similar protein loading by Coomassie staining, the mutant has less PMA antigen (α-CtAHA) and less active PMA (α-p^T947^). Densitometric analysis (ImageJ program) of three experiments like the one of Figure 7A indicate that the amount of PMA antigen in *sbt4.13-1D* mutant is 45 ± 7 % (mean ± standard error) of wild type and the level of phosphorylated and most active PMA is 43 ± 6 % of wild type. These differences are statistically significant with *p* < 0.01 by Student’s test. Therefore, the activated state of PMA is not changed in the *sbt4.13-1D* mutant but there is less enzyme protein. This is corroborated by measurements of ATP hydrolysis specific of PMA (Figure 7B), which shows that activity in the mutant is about 60% of wild type. The difference with quantification of PMA protein (Western blot) may be due to some unspecific ATP hydrolysis by contaminating phosphatases.

One explanation for the decreased amount of PMA in the *sbt4.13-1D* mutant is that the enzyme could be attacked by the over-expressed SBT4.13 protease. This mechanism was supported by transient expression of SBT4.13 in leaves of *Nicotiana benthamiana* by agro-infiltration, where the endogenous PMA was rapidly degraded (1–2 days) by the expressed protease (Figure S4).

**Figure 8:**
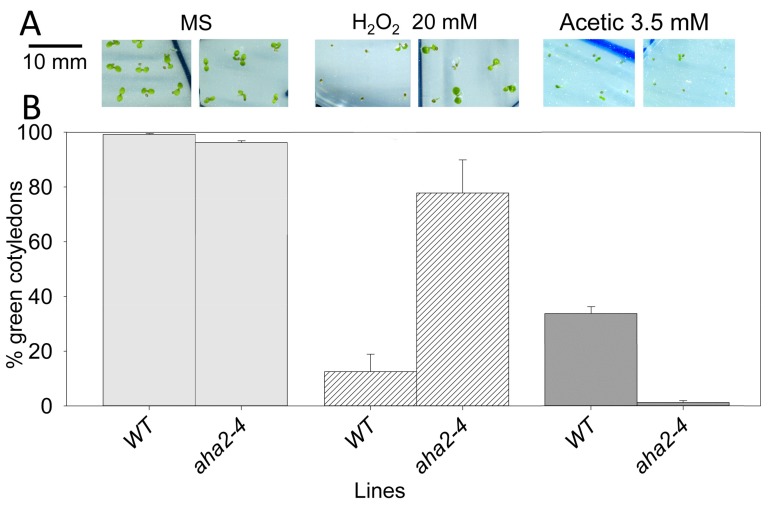
The *aha2-4* mutant is tolerant to H_2_O_2_ but sensitive to acetic acid. Seedlings of wild type (WT) and *aha2-4* mutant were germinated and grown for six days in normal MS medium and in medium containing H_2_O_2_ and acetic acid as indicated. (**A**): representative seedlings. (**B**): statistical data of green and expanded cotyledons from three experiments, each one with 30–40 seedlings from every line and condition. Error bars correspond to standard error and differences with wild type in medium containing H_2_O_2_ and acetic acid are statistically significant with *p* < 0.01 by Student’s t-test.

In order to check what phenotypic features of *sbt4.13-1D* can be explained by reduction of PMA activity, we tested the *aha2-4* mutant, which contains about 50% PMA activity of wild type [20]. As described at the latter reference, less PMA activity in *aha2-4* confers tolerance to toxic cations such as lysine, arginine, hygromycin, cesium and lithium, probably because of decreased membrane potential, as described at Section 2.3. Therefore, the observed decrease of PMA in *sbt4.13-1D* explains its tolerance to toxic cations (Figure 4). Also, as shown in Figure 8, the *aha2-4* mutant is tolerant to oxidative stress produced by H_2_O_2_, suggesting that the decreased content of PMA of *sbt4.13-1D* explains its tolerance to H_2_O_2_ (Figure 4). What cannot be explained by PMA decrease is the tolerance to organic acids of *sbt4.13-1D* because, as expected, *aha2-4* is very sensitive to organic acids (Figure 8). Therefore, the acid tolerance of the *sbt4.13-1D* mutant remained unexplained.

### 2.5. Intracellular Acidification Induces ROS Production by RBOH-D,F in Wild Type but not in sbt4.13-1D and the Atrboh-D,F Mutant is Acid Tolerant

In order to get clues about the mechanism of acid tolerance of the *sbt4.13-1D* mutant we performed a transcriptomic analysis looking for differentially expressed genes between mutant and wild-type plants. We found 101 genes expressed in the mutant, more than double the number in wild type, and 23 genes expressed in the wild type, more than double that in mutant. Most important functional categories were metabolism, stress responses (not including acid or proton stress) and hormone responses (Supplementary Data Set S1). No indication for a mechanism of acid tolerance could be found.

**Figure 9:**
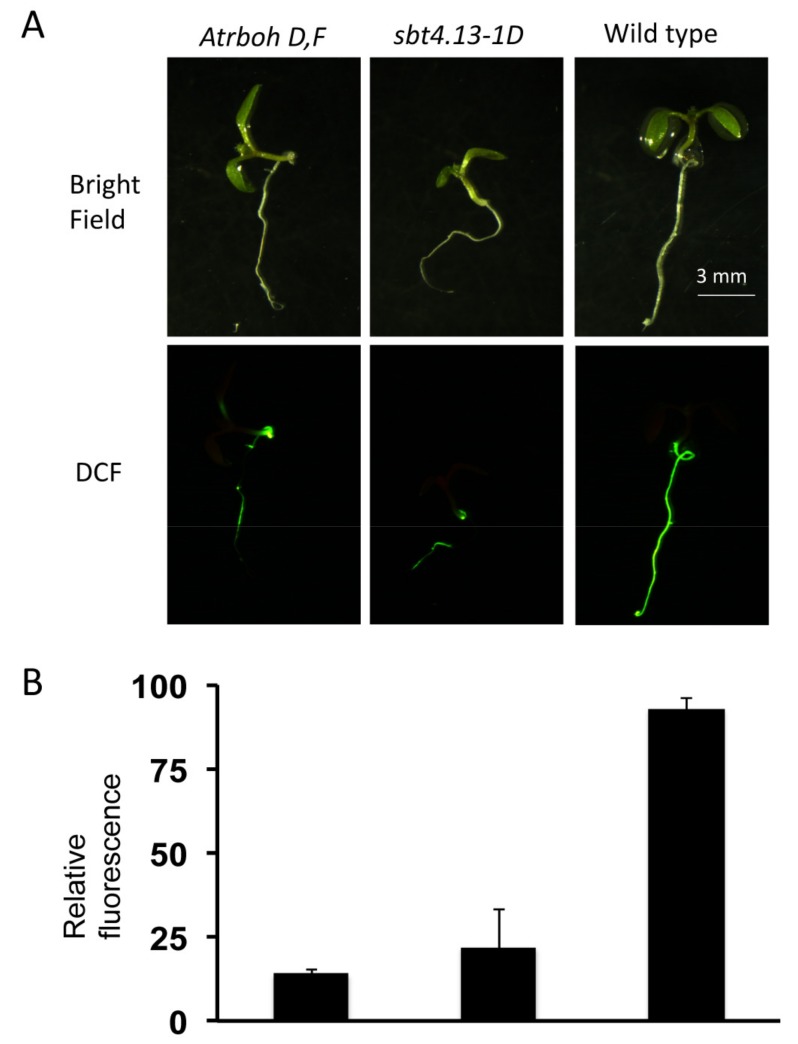
Acetic acid triggers production of ROS in wild type, but *sbt4.13-1D* and *atrboh-D,F* mutants have an attenuated response. (**A**) Bright field (upper panels) and fluorescence of 2′,7′-Dichlorofluorescein (lower panels) monitored by microscopy in six-day-old seedlings after 24 h incubation with 2.5 mM acetic acid (pH 5.5) and 30 min with 50 µM H_2_-DCFDA, as described in methods. (**B**) Quantification of relative fluorescence from 10 seedlings like those of part A performed with the program ImageJ. All the differences with wild type are statistically significant at *p* < 0.01 (Student’s t-test). The fluorescence of control plants not treated with acid was undetectable in all cases. Points of strong fluorescence were discarded as artefacts of handling the plantlets.

One working hypothesis we advanced at this stage was that intracellular acidification inhibits growth by activating production of some inhibitory molecule and that the *sbt4.13-1D* mutant is acid-tolerant by having a decreased production of this growth inhibitor. As many abiotic stresses induce production of ROS [21] we assumed that the acid-induced inhibitory molecules could be ROS. NADPH oxidases (NOXs, RBOHs) are plasma membrane, ROS-generating proteins [22] that could be degraded by over-expressed SBT4.13. Therefore, we tested the hypothesis that under our conditions intracellular acidification may activate NOXs to inhibit growth. Two NOXs highly expressed in *Arabidopsis* seedlings are RBOH-D and RBOH-F [22]. These oxidases are partially redundant as mediators of ABA-induced stomatal closing and therefore we used the double mutant *Atrboh-D,F* [23] to check for induction of ROS production by acids and for tolerance to acids. As indicated in Figure 9, ROS production is induced by acetic acid in seedlings of wild type but much less in *Atrboh-D,F* and *sbt4.13-1D* mutants (15% and 23% of wild type). This suggest that intracellular acidification induces ROS production largely dependent on RBOH-D,F.

**Figure 10:**
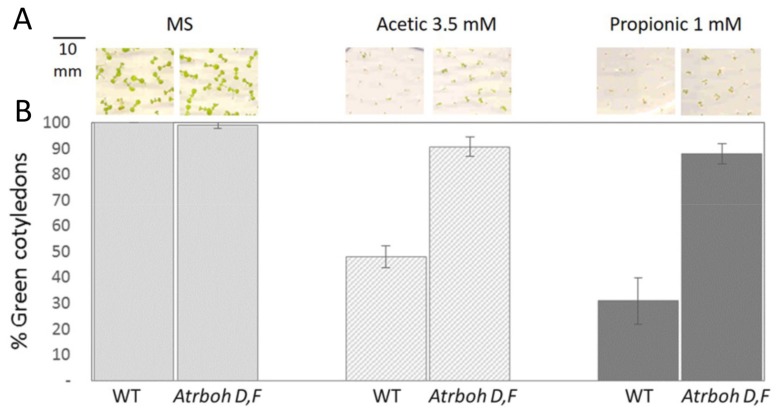
The *Atrboh-D,F* double mutant is tolerant to organic acids. Upper panels (**A**) show representative seedlings and the lower graphs (**B**) are statistical data of green and expanded cotyledons from three experiments, each one with 30–40 seedlings from every line and condition. Error bars correspond to standard error, and differences with wild type in medium containing acetic and propionic acids are statistically significant with *p* < 0.01 by Student’s t-test.

Another important result was that the *Atrboh-D,F* mutant has similar acid tolerance to the *sbt4.13-1D* mutant (Figure 10). Therefore, our final interpretation of the phenotype of the *sbt4.13* mutant was that over-expression of the subtilase decreases the activity of two plasma membrane proteins: PMA and acid-activated RBOH-D,F. The effect on PMA is negative for acid tolerance but it is compensated in the *sbt4.13-1D* mutant by inhibition of the acid-activated RBOH-D,F. This suggests that ROS production by these oxidases upon intracellular acidification is a major cause of inhibition of growth during seedling establishment.

We tested if inhibition of acid-activated RBOH-D,F is due to degradation of the oxidases by immunodetection with specific antibody against a C-terminal peptide conserved in D and F oxidases (described in reference [24]). The results (Figure S5) indicate that, after correcting the scanned pixels (ImageJ program) of the oxidase band for total protein load, no significant decrease of oxidase protein (less than 15%) occurs in the *sbt4.13-1D* mutant. Therefore, the inhibition of acid-activated ROS production by over-expression of SBT4.13 must occur by a mechanism different from degradation of the oxidases.

## 3. Discussion

### 3.1. A novel Arabidopsis Mutant Tolerant to Acids Over-Expresses Subtilase SBT4.13 and Has Less Plasma Membrane H^+^-ATPase (PMA)

In this work we screened activation-tagging lines of *Arabidopsis thaliana* to select genes that by over-expression confer tolerance of seedling growth to intracellular acidification produced by weak organic acids. This approach could identify limiting factors for pHc homeostasis involved in either H^+^ transport or toxicity [16,17]. The *sbt4.13-1D* mutant over-expresses subtilase gene *SBT4.13* about 100-fold and is acid tolerant. The mechanism of acid tolerance of this mutant was puzzling because is not based on improving H^+^ and K^+^ transport to avoid pHc acidification, as in previous mutants identified in our laboratory [16,17]. To our surprise, root epidermal cells of the *sbt4.13-1D* mutant have lower pHc and lower plasma membrane electrical potential (Em) than wild type. This was explained by decreased content and activity of PMA in the mutant, probably due to degradation of the proton pump by the over-expressed SBT4.13 protease. The *sbt4.13-1D* mutant exhibits a PMA activity about 60% of wild type, similar to that of the *aha2-4* mutant, a null mutant in one of the two major PMA isoforms of *Arabidopsis* [20]. The latter mutant is more sensitive to acids than wild type (Figure 8) and reduction of PMA content in *sbt4.13-1D* does not explain acid tolerance.

### 3.2. Intracellular Acidification Induces ROS Production Mediated by NADPH Oxidases (NOX), Explaining Growth Inhibition by Organic Acids

The acid tolerance of the *sbt4.13-1D* mutant can be explained by decreased toxicity of intracellular acidification, which is mediated by ROS production. The evidence is that a mutant without two major redundant NOXs (*Atrboh-D,F*) is acid tolerant and exhibits greatly reduced production of ROS during treatment with organic acids. These phenotypic features are shared by the *sbt4.13-1D* mutant and suggest that: (a) acid pHc activates NOXs and the produced ROS mediates growth inhibition; (b) the activity of RBOH-D,F oxidases is reduced by the over-expressed protease; (c) the poor pHc homeostasis of *sbt4.13-1D* mutant (due to decreased PMA) is compensated by decreased ROS production during acid treatment.

This mechanism of acid toxicity based on ROS production is relevant because knowledge about cellular targets of pHc that regulate growth in the absence of cell death is very limited [15,25,26]. There are indications that low pHc induces protein denaturation to inhibit growth [17] and that low pHc inhibits the growth-promoting protein kinase TORC1 in yeast [27] and carcinoma cells [28]. Also, cytoplasmic acidification inhibits protein synthesis required for growth and proliferation in BALB mouse cells [29]. Finally, acidic growth media induce ROS and antioxidant defenses in citrus roots [30] and animal cells [31,32], and in mammalian astrocytes acidic media activate NOX [32]. However, acid-tolerant mutants affected in relevant genes have not been described and mutants tolerant to acid pHc with decreased ROS production have not previously been reported.

### 3.3. PMA and a Protein-Mediating Acid Activation of NOX are Probably Degraded by the Over-Expressed Subtilase of the sbt4.13-1D Mutant

As there is no change in expression of PMA and NOX genes in the *sbt4.13-1D* mutant (Supplementary Data Set S1), one plausible mechanism for the reduction of PMA (Figure 7) and acid-activated NOX by over-expression of the SBT4.13 protease is degradation of PMA and of an unknown protein-mediating acid activation of NOXs. SBT4.13, as most subtilases, has a signal peptide at the N-terminus (MATLAASSSLLSCLLVLFLSSVSA) for secretion into endoplasmic reticulum (ER) (see SBT4.13 sequence at www.arabidopsis.org). Although most subtilases localize to the cell wall [33], we determined that an over-expressed fusion of SBT4.13 with GFP (Green Fluorescent Protein) is also at internal vesicles, probably ER (Figure S6). The loops of PMA facing the external side of the plasma membrane would also be exposed to the inside of ER and the over-expressed protease could attack these enzymes in all these locations. The recognition sites of SBT4.13 involve one or two basic residues (Lys, Arg) [34] and these amino acids are enriched in the external loop of PMA between trans-membrane helices 3 and 4 (267-RRKYRDGIDN-276, see AHA2 sequence at www.arabidopsis.org). In the case of RBOH-F, basic amino acids are enriched in the loops between trans-membrane helices 1 and 2 and between 3 and 4 [35], but no evidence of degradation could be obtained (Figure S5). Decreased PMA and ensuing depolarization of plasma membrane could reduce NOX activity because these enzymes are electrogenic (extrude electrons outside cells) and are activated by membrane potential negative inside [36,37]. However, the *aha2-4* mutant is not tolerant to acids (Figure 8) and therefore some additional mechanism must operate in the *sbt4.13-1D* mutant to reduce acid-activated NOX activity. One plausible mechanism is that over-expressed SBT4.13 could degrade some protein mediating the effect of acid pHc on NOX enzymes.

SBT4.13 is one of the subtilases that process the precursor of the hormonal peptide IDA (Influorescence Deficient in Abscission) [34]. However, we consider unlikely that this physiological role of SBT4.13 is responsible for the phenotype of the *sbt4.13-1D* mutant, mostly because this phenotype can be mimicked by genetically reducing the amounts of PMA and NOX. The acid tolerance phenotype is explained because reduced RBOH-D,F activity in the *sbt4.13-1D* mutant would decrease acid-induced ROS production. Tolerance to toxic cations is explained because reduced PMA in the *sbt4.13-1D* mutant would decrease the plasma membrane electrical potential, which contributes to the driving force for cation uptake (see Section 2.3). Tolerance to H_2_O_2_ can also be explained by decreased PMA and the resulting fall of pHc because oxidation of sulphydryl groups in glutathione and intracellular proteins is inhibited at acidic pH, when the most reactive thiolate form of sulphydryls is decreased [38]. The IDA hormonal peptide could participate in the diverse transcriptional response to SBT4.13 over-expression.

### 3.4. Regulation of NADPH Oxidases by pH

Cytosolic pH [25,26,39] and NOX enzymes [40,41,42,43,44] have both complex regulations and multiple physiological roles and we found a connection between these two important actors. Future studies should identify the mechanism of activation of NOXs by low pHc and the cellular targets regulating growth.

Known regulation of NOX enzymes in plants occurs by phosphorylation and through increase of cytosolic free calcium, the latter acting both directly on EF motifs of the oxidases and through Ca^2+^ sensor-activated kinases [41,42]. The in vivo activation of NOX by intracellular acidification identified in the present work could occur by either direct effect of acidic pH on the enzymes or by an increase of free cytosolic Ca^2+^ triggered by intracellular acidification, as described by Felle [45]. The role of intracellular free Ca^2+^ in activation of NOX and inhibition of seedling growth by acids is currently under investigation.

In animal cells NOXs are associated with an H^+^ efflux channel for electrical balance during NADPH oxidation and electron transport to outside cells [46]. This H^+^ channel would be activated by acidic pHc and could stimulate electrogenic NOX. Plant NOXs do not have these channels [22,40], probably because plant cells have an electrogenic plasma membrane PMA for electric balance during electron transport out of the cell. As PMA is activated by acidic pHc [16,19], this could contribute to NOX stimulation by hyperpolarization [36,37], but this hyperpolarization does not occur in the *sbt4.13-1D* mutant (Figure 6).

### 3.5. A Novel Pathway for Growth Inhibition by Organic Acids through Activation of NOX

A basal level of ROS is essential for life and is required for cellular proliferation and differentiation and for adaptation to biotic and abiotic stresses. On the other hand, high ROS levels, resulting from different stresses, trigger programmed cell death [2,3,4,21,47]. In our work mild intracellular acidification induced by low concentrations of organic acids produces an intermediate level of ROS that impairs growth without inducing cell death. This intermediate situation is considered in Figure 1 of the review of Petrov et al. [21], but most discussions in the field only refer to proteins that participate in the control of programmed cell death induced by high ROS levels [21]. Accordingly, there is little information about proteins that participate in growth inhibition by low ROS levels in plants.

Our future work will concentrate on approaches to identify these novel ROS targets. One possibility is that some proteins participating in ROS-triggered cell death also regulate ROS-triggered growth inhibition. However, known proteins controlling cell death are not related to cell growth but to programmed cell death, ROS production and stress responses [21]. One approach to novel ROS targets could be screening of activation-tagging mutant collections of *Arabidopsis* for tolerance of seedling growth to low H_2_O_2_ concentrations. However, available collections do not cover the whole genome and our exhaustive screenings for acid tolerance have provided a limited number of genes. A more convenient mutant collection, recently available, is provided by the *Arabidopsis* FOX lines developed by RIKEN, with each line over-expressing one complete cDNA [48]. A chemical approach based on mapping of sulfenic acid (in oxidized cysteine) modified proteins [49] could also identify oxidation targets. A promising novel approach could be exploiting natural variation in organic acid tolerance between *Arabidopsis* cultivars with sequenced genomes. These Genome Wide Association Studies (GWAS) [50,51] are underway in our laboratory.

## 4. Materials and Methods

### 4.1. Plant Materials and Growth Conditions

*Arabidopsis thaliana* wild type (ecotype Columbia Col-2; N907) and the H^+^-ATPase mutant *aha2–4* [52] (SALK082786), were obtained from Nottingham *Arabidopsis* Stock Centre (NASC; https://twitter.com/NascArabidopsis). The double mutant *Atrboh-D,F* [23] was obtained from Julian I. Schroeder and June M. Kwak, Division of Biological Sciences, University of California at San Diego, La Jolla, USA. The T-DNA activation-tagging seed collection (donated by W. Sheible and C. Sommerville) was obtained from NASC (code N31100).

Growth of wild type and derived mutants in greenhouse and in vitro culture was as described [18]. Briefly, the growth medium for in vitro culture (referred as MS medium) contained 0.4% Murashige and Skoog salts, 1% sucrose and 10 mM MES (2-N-morpholino)-ethanesulfonic acid) buffer adjusted to pH 5.5 with Tris base. The acetic (Ref. 131008, Applichem, Darmstadt, Germany), propionic (Ref. P1880, Sigma-Aldrich. St. Louis, MO, USA) and sorbic (Ref. S1626, Sigma-Aldrich) acids, as well as norspermidine (Ref.I1006, Sigma-Aldrich) were adjusted to pH 5.5 with Tris base or HCl before addition to media. LiCl (Ref. 25009) was from VWR International, Radnor, Pennsylvania, USA. Seedlings were grown for six days in Petri dishes for acid tolerance studies. *Arabidopsis* lines were propagated in pots containing 1:2 vermiculite/soil mixture. Long day illumination was used (16 h light/8 h dark, 23 ± 2 °C and 70 ± 5% relative humidity) for sterile plates in growth chambers and for pots in greenhouse. Wild type and mutants were propagated and seeds collected under identical conditions.

### 4.2. Isolation and Genetic Characterization of the Wat2-1D Mutant

The T-DNA activation-tagging seed collection from W. Sheible and C. Sommerville (see above) was utilized previously by our laboratory in partial form [16] and now was totally screened (63,000 lines) following the same methodology. Briefly, about 600,000 seeds were screened at high density (about 2000 seeds per 9 cm plate) on MS medium with 7 mM acetic acid. After six days, mutants with fully expanded green cotyledons were selected and grown in soil to collect seeds. Next generation was analyzed in a secondary screening at low seed density (about 150 seeds per plate) with 3.5 mM acetic acid and the mutant with stronger phenotype was named *wat2-1D* and further investigated. Genetic characterization and co-segregation analysis were as described [16].

### 4.3. Molecular Characterization of the sbt4.13 Mutant

Oligonucleotide primers utilized in the present work are shown in Table S1 and the following methods were described in references [16,18]: Southern-blot analysis to determine the number of copies of T-DNA insertions (hybridization probe amplified with primers 35S_F and 35S_R), plasmid rescue to sequence flanking regions of inserted DNA (primers 5′LB and 5′RB), PCR to detect T-DNA (primers 35S_F and 35S_R), determination of gene expression levels by qRT-PCR (primers At5g59120for and At5g59120rev, At5g59130for and At5g59130rev; At5g59120for and At5g59120rev) and standard sequencing to check constructions (primers M13direct, T7, SBT3, SBT4).

### 4.4. Construction of Transgenic Plants to Recapitulate the Mutant Phenotype

The *Arabidopsis* full-length cDNA clone corresponding to *SBT4.13* (At5g59120; code RAFL06-89-B10) was obtained from RIKEN BioResource Research Center (Wako, Saitama, Japan) [53,54] in plasmid pUNI51, a pBluescript derivative. This cDNA was amplified by PCR with primers ORFfor and ORFrev+stop (Table S1). PCR products were cloned into the pCR8/GW/TOPO plasmid (Invitrogen brand of Thermo Fisher Scientific, Waltham, Massachusetts, USA) and then recombined to binary plasmid pMDC32 [55], using Gateway technology and the LR Clonase reaction. This construction will express *SBT4.13* from a 2x35S promoter. Amplification and cloning first in pCR8 and then in pMDC83 [55] was also made with primers ORFfor and ORFrev-stop to express a fusion protein SBT4.13-GFP (Green Fluorescent Protein). Constructions in pMDC32 and pMDC83 were sequenced with primers MDCfor and MDCrev (Table S1). All recombinant binary plasmids were introduced into *Agrobacterium tumefaciens* GV3101::pMP90RK to transform *Arabidopsis*.

### 4.5. Transient Expression of SBT4.13-GFP in Nicotiana Benthamiana Leaves

The *SBT4.13* cDNA was amplified with primers ORFfor and ORFrev+stop (Table S1) and the PCR product cloned into pCR8/GW/TOPO and then recombined into binary plasmid pSPYNE-Kan [56] and introduced into *Agrobacterium tumefaciens* GV3101::pMP90RK (see above, Section 4.5). Infiltration of *Nicotiana benthamiana* leaves with *Agrobacterium* was as described [56,57].

### 4.6. Measurement of ROS Content in Plants with H2DCFDA

The fluorescein derivative dichlorodihydrofluorescein diacetate (H2DCFDA, Sigma-Aldrich) was utilized to detect ROS (H_2_O_2_, OH·) in plant tissues [58]. *Arabidopsis* seedlings were grown for six days in solid MS medium and then were transferred to liquid MS medium with and without 2.5 mM acetic acid. After 24 h, 50 µM H_2_DCFDA was added to the bathing medium and further incubated for 30 min. Plantlets were then photographed with a Leica MacroFluo macroscope system, as bright field and fluorescence (with a GFP filter). The JAVA-based image-processing program ImageJ (http://rsb.info.nih.gov/ij) was utilized for relative quantification of fluorescence.

### 4.7. Transcriptomic Analysis by Microarrays

These methods were described in [17]. Supplementary Data Set S1 shows genes induced and repressed in seedlings of the *sbt4.13-1D* mutant with respect to those of wild type *Arabidopsis* (Col-2) after growing in normal medium for six days. Functional categories were from Gene Ontology (www.geneontology.org). Original data were submitted to the MIAME-compliant database GEO (https://www.ncbi.nlm.nih.gov/geo/query/acc.cgi?acc=GSE142459; reviewer access during private period: wtsjueogtrqljkb).

### 4.8. Other Methods

Electrophysiological measurements of membrane potential and cytosolic pH were made in root epidermal cells from seedlings grown in vertical plates for 12-14 days by using H^+^ selective microelectrodes as described previously [13,16,17]. Determination of PMA ATPase activity in partially purified plasma membrane vesicles and immunoblot analysis of PMA (Western blot) were as described [13], but plantlets were grown in vertical plates instead of hydroponic culture. Visualization of SBT4.13-GFP fusion by fluorescence microscopy and plasmolysis to separate plasma membrane from cell wall was as in [18]. The antibody against RBOH-D,F was purchased from PhytoAB Inc. (San José, CA, USA).

## 5. Conclusions

To conclude, the pathway identified in the present work, from intracellular acidification to ROS generated by NOX and growth inhibition could be important for fundamental biology and for agriculture. Many stress conditions experienced by crops result in intracellular acidification and reinforcement of ROS targets could be one strategy to improve yields.

## Supplementary Materials

Supplementary materials can be found at https://www.mdpi.com/1422-0067/21/3/1173/s1.
