# Supplementary material for: An Arabidopsis Mutant Over-Expressing Subtilase SBT4.13 Uncovers the Role of Oxidative Stress in the Inhibition of Growth by Intracellular Acidification
Source: Int J Mol Sci. 2020 Feb 10;21(3):1173. doi: 10.3390/ijms21031173 (PMC7037345; doi:10.3390/ijms21031173)
Supplement: Supplementary file 1 [file ijms-21-01173-s001.zip › zipcosa/SuppFig.docx.zip!/SuppFig.docx]

Figure S1. The 109 and 68 mutants have a similar single insertion of T-DNA by Southern analysis. Genomic DNA was prepared from wild type and six acid tolerant candidates, digested with *Bam* HI, and hybridized with a probe for the upstream activating sequence (enhancer) of the 35S promoter. The 109 and 68 mutants are highlighted with red arrows. The position of two size markers is shown at the right black arrows.

Figure S2. 32 F2 plants from the cross of *wat2-1D* x Col2 (wild type, pollen) that are acid tolerant have T-DNA insertion. The 0.37 kb fragment of the upstream activating sequence (enhancer) of 35S promoter was detected by PCR and shown between two size markers of 0.4 and 0.3 kb (at the left). No band appears using DNA of wild type (run separately).

Figure S3. Germination and appearance of green and expanded cotyledons 6 days after sowing in normal MS medium of wild type (Col2 ecotype), *wat2-1D* mutant and recapitulation lines R5 and R7. All lines have more than 95% green and expanded cotyledons but the three *sbt4.13-1D* mutants have smaller size.

Figure S4. Immunodetection of PMA in leaves of *Nicotiana benthamiana* at different times after agro-infiltration with a plasmid expressing SBT4.13 from the 35S promoter. Partially purified preparations of plasma membrane vesicles were employed (see Methods) and 10 µg protein were applied per lane. Left panel corresponds to western blot with specific antibody against Arabidopsis PMA (cross-reacting with *Nicotiana* PMA). Right panel corresponds to staining of total protein with Coomassie R-250. The experiment was repeated twice with similar results.

Figure S5. NADPH oxidases RBOH-D and RBOH-F are not significantly decreased in the *sbt4.13-1D* mutant. Left panel:_immuno-detection with specific antibody against D and F oxidases in partially purified plasma membranes. The immuno-detected band at about 100 kDa corresponds to the oxidases. Right panel: staining of total protein with Direct Blue 71. The experiment has been repeated twice with similar results.

Figure S6. Detection of GFP in leaf epidermal cells of transgenic Arabidopsis plants expressing a fusion protein of SBT4.13 and GFP. Tissue samples were plasmolyzed by incubation with 1 M mannitol on glass slides for 1 h at room temperature. Fluorescence of GFP was observed by a Leica TCS-SL confocal microscope and laser scanning confocal imaging system. A 488 nm excitation wavelength and a 510 nm emission wavelength were used. When the plasma membrane (PM) separates from cell wall a substantial amount of the fluorescence detach from the cell wall and must occur in internal vesicles, probably endoplasmic reticulum.
