# Supplementary material for: An Arabidopsis Mutant Over-Expressing Subtilase SBT4.13 Uncovers the Role of Oxidative Stress in the Inhibition of Growth by Intracellular Acidification
Source: Int J Mol Sci. 2020 Feb 10;21(3):1173. doi: 10.3390/ijms21031173 (PMC7037345; doi:10.3390/ijms21031173)
Supplement: Supplementary file 1 [file ijms-21-01173-s001.zip › zipcosa/Table S1.docx.zip!/Table S1.docx]

**Supplementary Table S1. Oligonucleotide primers utilized in the present work**

| Name | Use | Sequence (5’🡪3’) |
| --- | --- | --- |
| 5’LB | Sequencing | AGATTTCCGAATTAGAATAA |
| 5’RB | Sequencing | GAAATGGATAAATAGCCTTGC |
| 35S_F | PCR | CAACATGGTGGAGCACGACA |
| 35S_R | PCR | GGGATCTAGATATCACATCA |
| At5g59110for | qRT-PCR | ACGAGCAACTTATGGAGCTGGTCA |
| At5g59110rev | qRT-PCR | AGTGCAAGGGTATCCGCAGTGTAA |
| At5g59130for | qRT-PCR | AAAGGGATCCTCACTGTGAACGCT |
| At5g59130rev | qRT-PCR | TTTGCTGACAAACTCACGGTTCGC |
| At5g59120for | qRT-PCR | ACCTGCCGCTGGTTTACTAACAGA |
| At5g59120rev | qRT-PCR | ATCAACCGCAATGGTGTTTGGACC |
| ORFfor | Cloning SBT4.13 | ATGGCGACGCTAGCAGCTTC |
| ORFrev+stop | Cloning SBT4.13 | TCAGTAATCACTAGTATAAACAACAATGGGAC |
| ORFrev-stop | Cloning SBT4.13 | GTAATCACTAGTATAAACAACAATGGGACTTC |
| M13 direct | Sequencing | TGTAAAACGACGGCCAG |
| T7 | Sequencing | TAATACGACTCACTATAGGG |
| SBT3 | Sequencing | CAGTTGTGGACGCAAGCTTCT |
| SBT4 | Sequencing | ACCTGCCGCTGGTTTACTAACAGA |
| MDCfor | Sequencing | AGGTAGTGGCGGGTCACG |
| MDCrev | Sequencing | CGAACTTTCTCCGGTGACC |
